# Expression Pattern and Function Analysis of AtPPRT1, a Novel Negative Regulator in ABA and Drought Stress Responses in Arabidopsis

**DOI:** 10.3390/ijms20020394

**Published:** 2019-01-17

**Authors:** Linsen Pei, Lu Peng, Xia Wan, Jie Xiong, Zhibin Liu, Xufeng Li, Yi Yang, Jianmei Wang

**Affiliations:** Key Laboratory of Bio-Resources and Eco-Environment of Ministry of Education, College of Life Sciences, Sichuan University, Chengdu 610065, China; guyuexiaosheng_1@163.com (L.P.); woshi_1221@163.com (L.P.); wanxia20181219@163.com (X.W.); nankaixiongjie@mail.nankai.edu.cn (J.X.); lzb2003@163.com (Z.L.); lixufeng0507@gmail.com (X.L.); yangyi528@scu.edu.cn (Y.Y.)

**Keywords:** AtPPRT1, negative regulator, ABA, drought, Arabidopsis

## Abstract

Abscisic acid (ABA) plays a fundamental role in plant growth and development, as well as in the responses to abiotic stresses. Previous studies have revealed that many components in ABA and drought stress signaling pathways are ubiquitinated by E3 ligases. In this study, AtPPRT1, a putative C3HC4 zinc-finger ubiquitin E3 ligase, was explored for its role in abiotic stress response in *Arabidopsis thaliana*. The expression of *AtPPRT1* was induced by ABA. In addition, the β-glucuronidase (*GUS*) gene driven by the *AtPPRT1* promoter was more active in the root hair zone and root tips of primary and major lateral roots of young seedlings in the presence of ABA. The assays for seed germination, stomatal aperture, root length, and water deficit demonstrated that the *AtPPRT1*-overexpressing Arabidopsis was insensitive to ABA and sensitive to drought stress compared with wild-type (WT) plants. The analysis by quantitative real-time PCR (qRT-PCR) revealed that the expression of three stress-inducible genes (*AtRAB18*, *AtERD10*, and *AtKIN1*) were upregulated in the *atpprt1* mutant and downregulated in *AtPPRT1*-overexpressing plants, while two ABA hydrolysis genes (*AtCYP707A1* and *AtCYP707A3*) were downregulated in the *atpprt1* mutant and upregulated in *AtPPRT1*-overexpressing plants in the presence of ABA. AtPPRT1 was localized in the mitochondria. Our findings indicate that AtPPRT1 plays a negative role in ABA and drought stress responses.

## 1. Introduction

As sessile organisms, plants face fluctuating environments that are often unsuitable for growth and development. These adverse environmental conditions include biotic and abiotic stresses, such as pathogen infection and herbivore attack, drought, heat, radiation, cold, high salt, and so on. Among these conditions, drought stress is considered the most serious environmental factor affecting the geographical distribution of plants, limiting plant productivity in agriculture and threatening food security [1,2]. It was reported that drought is occuring globally at a high frequency and intensity. Furthermore, the duration and intensity of droughts have generally increased globally [3,4]. Drought causes osmotic pressure and oxidative stress in plants and damages cellular components including membrane lipids, proteins, and nucleic acids. Interestingly, plants have developed complex and diverse mechanisms to cope with such situations. One important mechanism is the accumulation of abscisic acid (ABA), which in turn activates many adaptive responses in plants [5].

As a crucial phytohormone, ABA plays a vital role in many aspects of plants, including seed dormancy and germination, root growth, seedling development, and the adaptive response to environmental stresses such as drought, salt, cold, and other abiotic stresses [6,7,8,9]. It has also been reported that ABA is involved in plant pathogens by increasing plant susceptibility to pathogens, especially to fungi [10,11]. During the past decades, many ABA signaling components have been identified, such as ABA receptors, phosphatases, kinases, transcription factors, and ABA-regulated genes [9]. Among these components, there are several that are usually subjected to post-translational modifications, including phosphorylation, sumoylation, and ubiquitination [9,11,12,13].

Genome-wide studies have revealed that the ubiquitin-26S proteasome system (UPS) is an exceedingly large and complex route for protein removal, occupying nearly 6% of the Arabidopsis proteome [14]. Since its discovery in the late 1970s, UPS appears to be omnipresent in many research fields [15]. Ubiquitination can be the attachment of one or more single ubiquitin molecules (monoubiquitination) or of ubiquitin polymers linked internally through one of several Lys residues in ubiquitin itself (polyubiquitination) [14,16]. In the monoubiquitination system, the 76-amino acid protein ubiquitin acts as a covalent molecular tag, and its attachment requires three distinct enzymes that are referred to as E1 (ubiquitin-activating enzyme), E2 (ubiquitin-conjugating enzyme), and E3 (ubiquitin ligase) [17,18,19]. It is well known that the ubiquitinated protein, which is recognized by E3 ligase, is degraded by 26S proteasome.

E3 ubiquitin ligases, as central regulators in many cellular and physiological processes in plants, comprise a highly diverse and important group of enzymes. In Arabidopsis, the more than 1300 genes that are predicted to encode E3 ubiquitin ligases are classified into four different types: really interesting new gene (RING), U-box, homology to E6-AP C terminus (HECT), and Cullin (Cul)-RING ligases (CRLs) [15,20,21,22]. The RING domain was first identified in a protein encoded by the Really Interesting New Gene, the source of the domain name [23]. The RING-type E3 ubiquitin ligase, the most abundant E3 ubiquitin ligase family, is characterized by the presence of a cysteine-rich domain that coordinates two zinc atoms [20,24]. In Arabidopsis, 469 predicted proteins with RING domains have been identified using database searches followed by extensive manual management, and approximately 120 sequences coding the RING motif associated with one or more transmembrane domains have been found [20,22]. To characterize the E3 ubiquitin ligases and to identify their substrates are the two important objectives in this field [18].

It was reported previously that *SfIAP,* a homologous gene of Arabidopsis *AT1G68820* in insect (an inhibitor of apoptosis), enhances the tolerance of transgenic plants to several abiotic stresses [25]. *AT1G68820* encodes a protein with the RING domain and Tmemb_185A domain (abbreviated as AtPPRT1). In order to study the biological function of AtPPRT1 in Arabidopsis, we identified an *atpprt1* mutant and constructed *AtPPRT1*-overexpressing lines. The expression of *AtPPRT1* was induced by ABA and AtPPRT1 was localized in mitochondria. The qRT-PCR result shows that the expression of *AtPPRT1* was induced by ABA. We found that *AtPPRT1*-overexpressing lines showed a lower tolerance to drought and compared with WT plants, plants overexpressing *AtPPRT1* were insensitive to ABA. Our analysis demonstrates that AtPPRT1 acts as a negative regulator in ABA and drought stress responses.

## 2. Results

### 2.1. AtPPRT1 Encodes a Previously Uncharacterized Protein

**Figure 1:**
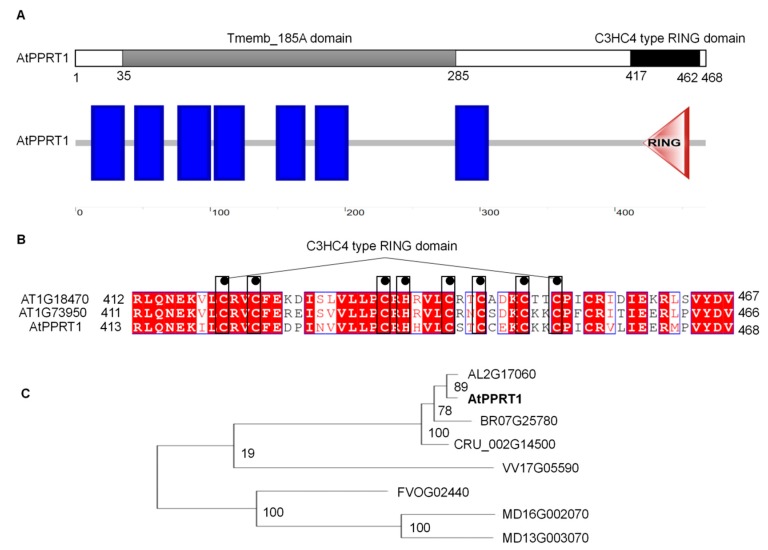
Domain organization and phylogenetic analysis of AtPPRT1. (**A**) Schematic diagram of AtPPRT1. The gray and dark bars in the upper line indicate the Tmemb_185A and RING (Really Interesting New Gene) domains. The blue boxes and red triangle in the lower line indicate the transmembrane and RING domains, respectively; (**B**) The RING domains of three Arabidopsis AtPPRTs. The amino acids of the RING domain were aligned using DNAMAN8.0; (**C**) Phylogenetic relationship of PPRTs and its homologs. Eight PPRT proteins from seven species were selected and analyzed by MEGA7.0. Bootstrap values from 1000 replicates are indicated at each node.

Insect *SfIAP*, a homologous gene of Arabidopsis *AT1G68820*, enhances the tolerance of transgenic plants to several abiotic stresses [25]. Hence, there was speculation that AT1G68820 is an apoptosis inhibitor in Arabidopsis [26]. According to the information about AT1G68820 in NCBI (https://www.ncbi.nlm.nih.gov/), TAIR (https://www.arabidopsis.org/), and PLAZA (https://bioinformatics.psb.ugent.be/plaza/versions/plaza_v3_dicots/), it was found that *AT1G68820* is comprised of 1407 base pairs encoding a 468 amino acid protein that contains a Tmemb_185A domain (amino acids 35 to 285) at the N-terminus and a C3HC4-type RING domain (amino acids 417 to 462) at the C-terminus. In PLAZA, there are 30 homologous genes of *AtPPRT1* in 18 dicots. Analysis of the multiple amino acid sequence alignment indicates that *AtPPRT1s* are highly conserved in many species, and four Cruciferous genes (*AL2G17060*, *AtPPRT1, BR07G25780*, and *CRU_002G14500*) exhibit high similarity (https://bioinformatics.psb.ugent.be/plaza/versions/plaza_v3_dicots/) (Figure 1C). In Arabidopsis, there are three homologs: AtPPRT1, AT1G73950, and AT1G18470 (Figure 1B).

### 2.2. Identification of Arabidopsis AtPPRT1 Mutant and AtPPRT1-Overexpressing Lines

**Figure 2:**
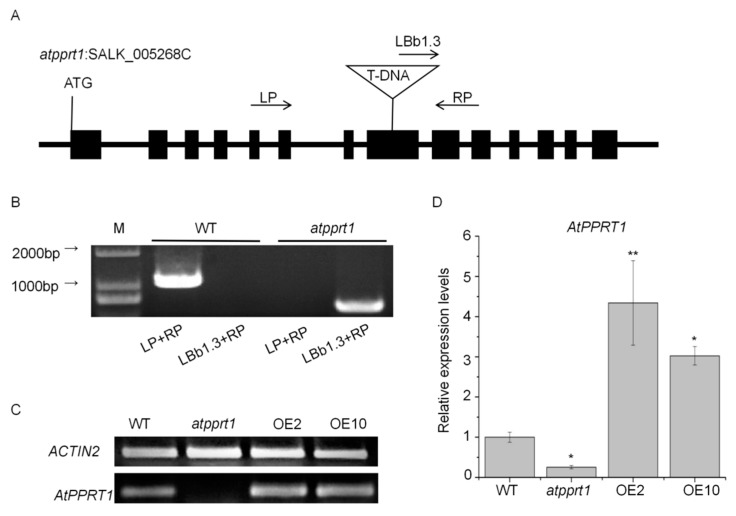
Identification of *atpprt1* mutant and *AtPPRT1*-overexpressing lines. (**A**) The structure of the *AtPPRT1* gene and T-DNA insertion site in the *atpprt1* mutant (SALK_005268C); (**B**) Molecular analysis of wild-type (WT) and the *atpprt1* mutant. The primers (LP, RP, and LBb1.3) were used in the experiment. M represents the molecular marker; (**C**) Semi-quantitative RT-PCR analysis of *AtPPRT1* expression levels in WT, *atpprt1*, and *AtPPRT1*-overexpressing lines OE2 and OE10; (**D**) qRT-PCR analysis of *AtPPRT1* expression levels in WT, *atpprt1*, and OE2 and OE10. ACTIN2 was used as the internal control for both semi-quantitative RT-PCR and qRT-PCR. The values are the average of three individual biological replications. Error bars represent ± SD (*n* = 3, * *p* < 0.05 and ** *p* < 0.01, *t*-test).

To investigate the function of *AtPPRT1*, we screened a homozygous *atpprt1* T-DNA insertion mutant (SALK_005268C) from ABRC (Arabidopsis Biological Resource Center; http://abrc.osu.edu/), and generated two independent *AtPPRT1*-overexpressing lines (abbreviated as OE2 and OE10). The *atpprt1* mutant and the insertion site of *atpprt1* was confirmed by genome PCR and sequencing, respectively (Figure 2B). A single copy of T-DNA was inserted in the *atpprt1* mutant and T-DNA was inserted in the eighth exon of *AtPPTR1* (Figure 2A). The results of semi-quantitative RT-PCR and real-time PCR showed that the transcripts of *AtPPRT1* were greatly reduced in the *atpprt1* mutant, and increased by different amounts in the OE lines compared with WT (Figure 2C,D).

### 2.3. The Expression of AtPPRT1 is Induced by ABA and is Increased in the Root Tips of Seedlings under ABA Treatment

**Figure 3:**
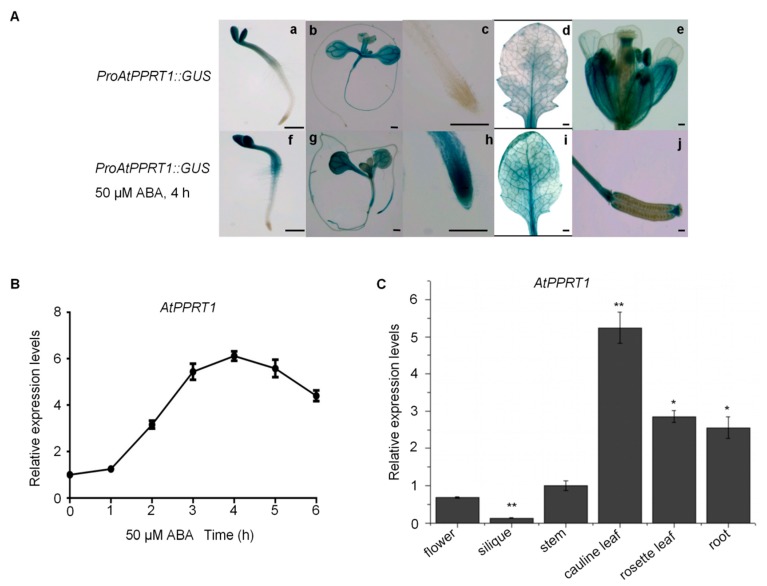
Tissue-specific expression of *AtPPRT1* and its ABA-induced expression levels. (**A**) Expression pattern of *AtPPRT1* in *ProAtPPRT1::GUS* transgenic Arabidopsis before (**a**–**e**,**j**) and after (**f**–**i**) ABA treatment. Tissues of *AtProPPRT1::GUS* transgenic plants including 3-day-old seedlings (**a**), 7-day-old seedlings (**b**), and its root tip of the main root (**c**), rosette leaf of a 3-week-old plant (**d**), flower (**e**) and silique (**j**) of a 40-day-old plant. The plant samples of (**f**–**i**) shared the same materials with those of (**a**–**d**), respectively, despite the former ones processed by 50 μM ABA for 4 h. Bars = 500 μm; (**B**) qRT-PCR analysis of *AtPPRT1* expression levels induced by ABA; (**C**) qRT-PCR analysis of *AtPPRT1* expression in different tissues of 40-day-old seedlings. The values are the average of three individual biological replications. Error bars represent ± SD (*n* = 3, * *p* < 0.05 and ** *p* < 0.01, *t*-test).

The seedlings of 7-day-old wild-type Arabidopsis were used to analyze the relative expression level of *AtPPRT1* in the presence of 50 μM ABA. The data show that the expression of *AtPPRT1* was induced by ABA and its expression was maximally activated after 4 h of ABA treatment (Figure 3B).

To explore tissue-specific expression of *AtPPRT1*, *ProPPRT1::GUS* constructs which comprised 1492 base pairs upstream of the ATG translation start codon of *AtPPRT1*, were transformed into wild-type Arabidopsis. The activity of the *AtPPRT1* promoter was determined using GUS histological staining. In the absence of ABA, the visible staining shows that *AtPPRT1* was expressed in cotyledons, hypocotyl of 3-day-old seedlings (Figure 3(A-a)), main leaf veins, hypocotyl of 7-day-old seedlings (Figure 3(A-b)), and the main vascular bundles of mature plant leaves (Figure 3(A-d)). It also was expressed in the reproductive organs, including the sepals, petals, stamens, rachis, and beak of siliques (Figure 3(A-e,A-j)). However, there was no GUS staining in the root tips of seedlings (Figure 3(A-c)) and immature seeds (Figure 3(A-j)).

When the transgenic plants were treated with 50 μM ABA for 4 h, the expression pattern of *AtPPRT1* had some changes in the young plants. For example, the promoter of *AtPPRT1* was more active in the root hair zone of 3-day-old seedlings (Figure 3(A-f)) compared with ABA-free seedlings (Figure 3(A-a)). High visible GUS staining was observed in the root tips of primary and major lateral roots (Figure 3(A-g,A-h)). Bioinformatics analysis revealed that MBS and G-BOX, cis-element response to ABA and drought, were found in the promoter of *AtPPRT1* (see Supplementary Figure S1). These results indicate that the expression pattern of *AtPPRT1* varies with the growth period and changes after ABA treatment.

In addition, mRNA levels of *AtPPRT1* displayed the highest expression in cauline leaves and the lowest expression in siliques (Figure 3C).

### 2.4. AtPPRT1 Acts as a Negative Regulator in Arabidopsis Response to ABA

**Figure 4:**
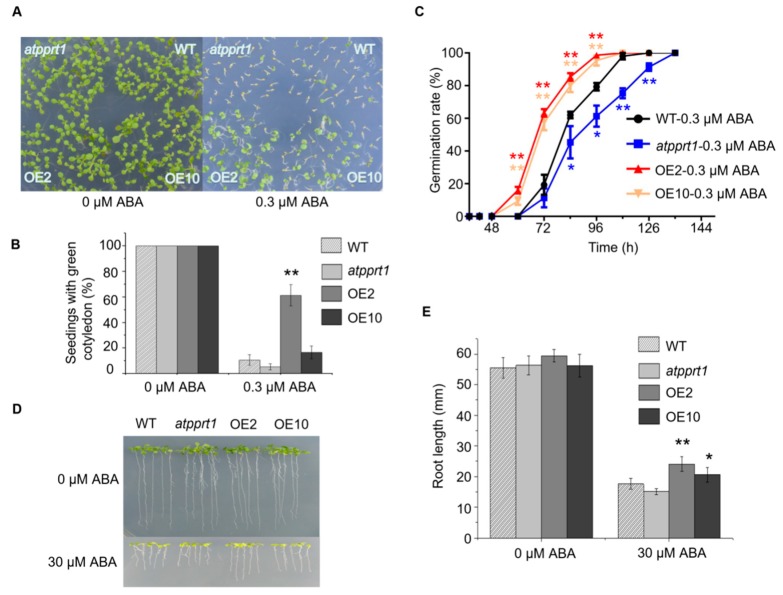
AtPPRT1 acted as the negative regulator in Arabidopsis signaling pathway. (**A**,**B**) Phenotype and green cotyledon rates of WT, *atpprt1*, OE2, and OE10 grown for 7 days on MS or MS supplemented with 0.3 μM ABA; (**C**) Germination rates of each line grown on MS supplemented with or without 0.3 μM ABA. Error bars represent ± SD (*n* = 50, * *p* < 0.05 and ** *p* < 0.01, *t*-test); (**D**,**E**) Phenotype and root length of WT, *atpprt1*, OE2, and OE10 planted 3 days on MS following 7 days vertical culture on MS or MS supplemented with 30 μM ABA. The values are the average of three individual biological replications. Error bars represent ± SD (*n* = 21, * *p* < 0.05 and ** *p* < 0.01, *t*-test).

**Figure 5:**
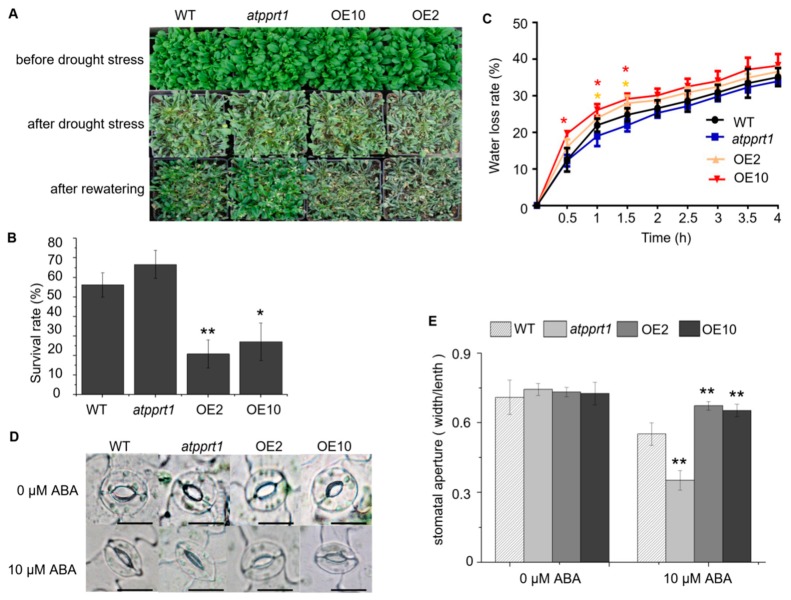
*AtPPRT1* negatively regulates drought stress response in Arabidopsis. (**A**,**B**) Phenotype and survival rates of WT, *atpprt1*, OE2, and OE10. Whole plants were subjected to drought stress for 13 days and then re-watered. Photos were taken 2 days after re-watering. Error bars represent ± SD (*n* = 3, * *p* < 0.05 and ** *p* < 0.01, *t*-test); (**C**) The water loss rates of WT, *atpprt1*, OE2, and OE10 under dehydration condition. Detached leaves from mature plants, exposed to air at room temperature and same humidity conditions, were weighed every 30 min; (**D**) Analysis of ABA-mediated stomatal closure. Detached leaves from mature plants were treated with or without 10 μM ABA for 2 h. Stomatal apertures were photographed using a fluorescence microscope (DMI6000B, Leica, Wetzlar, Germany); (**E**) Length/width ratios of stomas were measured and calculated using Image J. Bars represent 10 μm. The values are the average of three individual biological replications. Error bars represent ± SD (*n* = 100, * *p* < 0.05 and ** *p* < 0.01, *t*-test).

To confirm whether *AtPPRT1* is involved in the response to ABA, germination and seedlings with green cotyledons of WT, *atpprt1*, OE2, and OE10 were analyzed. The results show no obvious difference between WT, *atpprt1*, OE2, and OE10 in the absence of ABA. However, the OE lines were more insensitive to ABA. During germination, the OE lines germinated faster than WT, while *atpprt1* germinated slower than WT (Figure 4A,C). The rates of seedlings with green cotyledons of *atpprt1* mutants and WT were only around 4% and 10%, and that of OE2 and OE10 were 61% and 16% in the presence of ABA (Figure 4B,D,E). After 10 days of growth, the root lengths of all plants were recorded and analyzed. There was no significant difference in root lengths between the four lines on MS medium, while the root lengths of the OE lines were significantly longer than that of WT on MS containing ABA. To assess stomatal movement, rosette leaves were harvested from mature plants of WT, *atpprt1*, OE2, and OE10. The stomatal apertures reduced to 22% in WT, about 52% in *atpprt1*, 8% in OE2, and 10% in OE10 compared to plants without ABA treatment (Figure 5D,E). From the results, it was clearly demonstrated that *AtPPRT1* inhibited ABA-mediated stomata closure. These results indicate that the *AtPPRT1*-overexpressing lines displayed an insensitivity to ABA, suggesting that *AtPPRT1* acts as a negative regulator in Arabidopsis response to ABA.

### 2.5. AtPPRT1 Negatively Regulates Arabidopsis Response to Drought Stress

To assess the role of *AtPPRT1* in Arabidopsis drought stress response, WT, *atpprt1*, OE2, and OE10 plants were subjected to drought stress. The results show that nearly 66.6% of the *atpprt1* mutants could survive from dehydration, while the survival rates of OE2 and OE10 were reduced to 20.8% and 27%, respectively (Figure 5A,B). It was concluded that *AtPPRT1*-overexpressing lines were more sensitive to drought stress, while *atpprt1* plants displayed more tolerance to drought stress. After detachment for 1 h, the dehydration rates of WT, PPRT1, OE2, and OE10 were 25%, 21%, 28%, and 30%, respectively. The result shows that *AtPPRT1*-overexpressing plants lost water faster than WT (Figure 5C). In addition, the analysis of germination rates under mannitol stress revealed that the *atpprt1* mutant showed higher tolerance to mannitol compared with WT (Supplementary Figure S2). These results indicate that AtPPRT1 negatively regulates Arabidopsis response to drought stress.

### 2.6. AtPPRT1 is Localized in the Mitochondria

**Figure 6:**
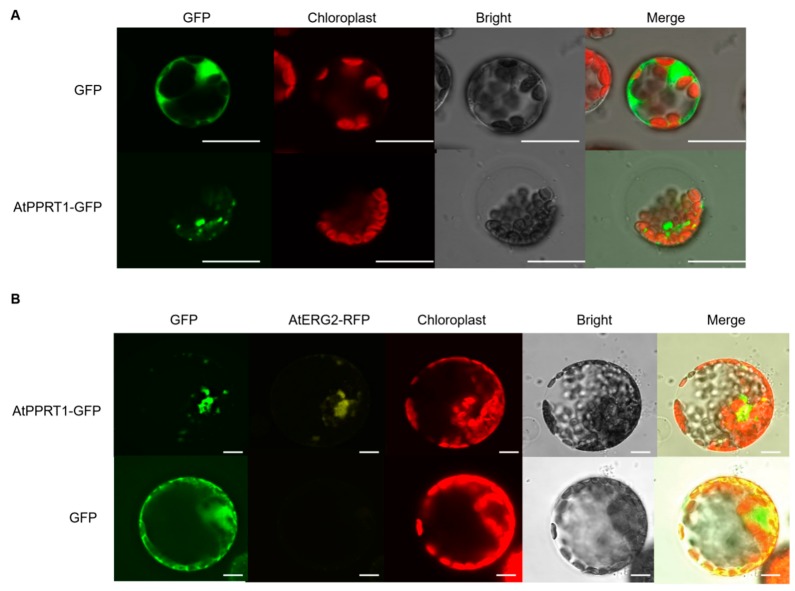
AtPPTR1 is localized in the mitochondria. (**A**) Subcellular localization of AtPPRT1 in Arabidopsis protoplast. The AtPPRT1 coding sequence was cloned into PBI221 containing a CaMV35S promoter for green fluorescence protein (GFP); (**B**) Subcellular localization of AtPPRT1 in tobacco protoplast. The AtPPRT1-GFP and AtERG2-RFP (a mitochondria marker) transiently co-transformed into tobacco protoplast. Auto-fluorescence of chloroplast and bright-field images are also shown for these transgenic cells. All images in this figure were obtained from one optic section. Scale bars are equivalent to 20 μm.

To investigate the subcellular localization of AtPPRT1, an AtPPRT1-GFP construct was transiently transformed into Arabidopsis mesophyll protoplasts. The results show that AtPPRT1-fused GFP displayed small spots in the field (Figure 6A). From website information (http://suba.live/suba-app/factsheet.html?id=AT1G68820), AtPPRT1 was predicted to localize in the mitochondria. Subsequently, the mitochondrial markers AtERG2-RFP and AtPPRT1-GFP were co-transformed into tobacco mesophyll protoplasts. The results confirm that AtPPRT1 was localized in the mitochondria (Figure 6A).

### 2.7. Disruption or Overexpression of AtPPRT1 Alters the Expression Levels of Stress-Inducible Genes and ABA Hydrolysis Genes

**Figure 7:**
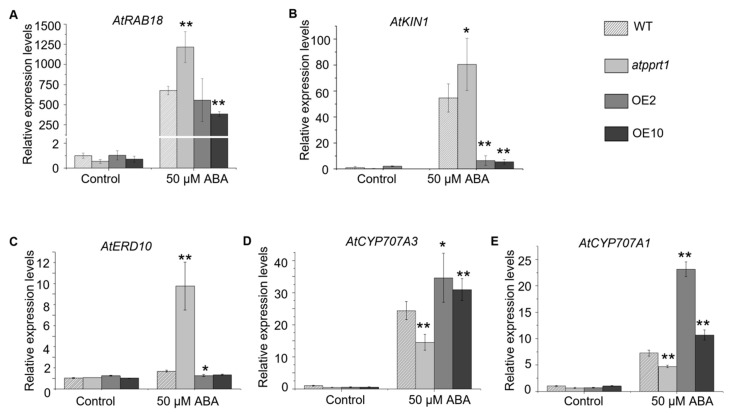
Transcriptional expression levels of stress-inducible genes and ABA hydrolysis genes before and after ABA treatment. Twelve-day-old seedlings were treated with or without 50 μM ABA for 4 h, and the transcriptional expression levels of *AtRAB18*, *AtERD10*, *AtKIN1*, *AtCYP707A1*, and *AtCYP707A3* were tested by qRT-PCR analysis. (**A**–**C**) qRT-PCR analysis of *AtRAB18 AtERD* and *AtKIN1* expression levels in WT, *atpprt1* and OE2 and OE10 treated with or without 50 μM ABA. (**D**) and (**E**) qRT-PCR analysis of *AtCYP707A1* and *AtCYP707A3* expression levels in WT, *atpprt1* and OE2 and OE10 treated with or without 50 μM ABA. The values are the average of three individual biological replications. Error bars represent ± SD (*n* = 3, * *p* < 0.05, ** *p* < 0.01, *t*-test).

In order to explore the roles of *AtPPRT1* in Arabidopsis response to abiotic stresses, three stress-inducible genes and two ABA hydrolysis genes were subjected to transcriptional expression analysis. In this study, 12-day-old seedlings were treated with 50 μM ABA for 4 h, and then the transcriptional expression levels of *AtRAB18*, *AtERD10*, *AtKIN1*, *AtCYP707A1*, and *AtCYP707A3* were analyzed by qRT-PCR. The expression levels of *AtRAB18*, *AtERD10*, *AtKIN1*, *AtCYP707A1*, and *AtCYP707A3* showed no differences in WT, *atpprt1*, OE2, and OE10 in the absence of ABA. Nevertheless, the expression levels of *AtRAB18* were significantly activated by ABA in all kinds of plants in the presence of ABA (Figure 7A). Furthermore, *AtRAB18* was induced more in *atpprt1* than in WT and OE lines. *AtKIN1* was dramatically activated in WT and *atpprt1*, whereas it was induced slightly in OE lines in presence of ABA (Figure 7B). Interestingly, *AtRED10* was upregulated around five fold in *atpprt1*, whereas there were no effects of ABA on OE lines and WT (Figure 7C). The expression levels of *AtRAB18*, *AtERD10*, and *AtKIN1* were inversely proportional to the expression of *AtPPRT1*. On the other hand, the expression of *AtCYP707A3* was upregulated only 15 fold in *atpprt1*, while it was upregulated around 23 fold in WT and around 33 fold in OE lines (Figure 7D). Similarly, the expression of *AtCYP707A1* was upregulated only 5 fold in *atpprt1*, but it was upregulated around 8 fold in WT, and more than 10 fold in OE lines (Figure 7E). The relative expression levels of *AtCYP707A1* and *AtCYP707A3* are consistent with expression levels of *AtPPRT1* in different plant materials. In summary, AtPPRT1 plays a negative role in drought stress response through upregulating the expression of stress-inducible genes such as *AtRAB18*, *AtERD10*, and *AtKIN1* and downregulating the expression of ABA hydrolysis genes such as *AtCYP707A1* and *AtCYP707A3* directly or indirectly.

## 3. Discussion

The RING-type E3 ligases, which could interact with and degrade specific substrates by ubiquitination, are involved in many cellular processes, such as transcription, signal transduction, recombination, rhizobial infection, nodule organogenesis, plant photomorphogenic responses, and plant disease resistance [15,27,28,29,30]. AtPPRT1 is a representative C3HC4-type RING protein encoded by *AT1G68820*, and it shares two homologs encoded by *AT1G73950* and *AT1G18740* (Figure 1B). AtPPRT1 and its homologs have a highly conserved representative RING-HCa domain with two amino acids between metal ligands 7 and 8 (Figure 1B). A previous study revealed that a large proportion of the RING proteins (more than 70%) that were selected to test ubiquitin activity was capable of mediating polyubiquitination [23]. Mutation of one or more of the metal ligands was shown to disrupt the ability of the RING domain to promote protein ubiquitination, and the RING domain was necessary for the E2-dependent protein ubiquitination [23,31]. In this study, truncated AtPPRT1 without any transmembrane domains did not show any E3 ligase activity [32]. It means that the E3 ligase activities of AtPPRT1 RING domain need its transmembrane domains for autoubiquitination or a specific target to finish the ubiquitination.

*AtPPRT1* was expressed mainly in vegetative organs in the absence of ABA, while the expression of AtPPRT1 was greatly induced in root tips of primary and major lateral roots in the presence of ABA. It is well known that ABA is synthesized in roots and leaves [12,33,34,35]. Guard cells, the main ABA target sites in terms of stomatal closure, autonomously synthesized ABA required for the stomatal response to leaf water status [35,36,37]. Our work also demonstrated that AtPPRT1 is involved in the expression of *AtCYP707A1* and *AtCYP707A3*, two cytochrome P450 monooxygenases. AtCYP707A3, a key catalytic enzyme in ABA hydroxylation, is predominantly expressed in vascular tissues and regulates the total amount of ABA accumulated in leaves [12,38]. High ABA levels have been detected in *cyp707a3* mutant plants even under high humidity conditions [39]. *AtCYP707A1*, which is preferentially expressed in guard cells, makes a minor contribution to the bulk ABA content in leaves, but regulates the stomatal aperture similarly to *CYP707A3* [39]. It had been concluded that site-specific ABA levels are determined based on a combination of biosynthesis, catabolism, and transport in vascular cells and guard cells [40,41]. Hence, AtPPRT1 is likely to participate in ABA metabolism. The sites of ABA synthesis and actions are often not clearly defined because cells in a relatively wide range of tissues and organs can synthesize hormones as well as respond to them [12].

Upregulation of *AtPPRT1* resulted in Arabidopsis being insensitive to ABA, such as higher seed germination potential, more seedlings with green cotyledon, longer root length, and larger stomatal aperture. Nevertheless, no significant physiological alteration was found between WT and *atpprt1* in the presence of ABA. The main reason for this phenomenon is that *AtPPRT1* has two homologous genes, which could have partially made up for the functional deficiency of AtPPRT1. Mutation of *AtPPRT1 (atpprt1* mutant) led to increased tolerance of plants to drought stress compared to *AtPPRT1*-overexpressing plants. Similar to the response to ABA, there was also no significant difference between WT and *atpprt1* in response to drought stress, indicating the functional redundancy of the homologous genes of AtPPRT1.

*RAB18*, *ERD10*, and *KIN1* are stress-inducible genes with similar functions and are expressed at higher levels following low temperature, exogenous ABA, and dehydration [42,43,44,45,46,47]. *RAB18* and *ERD10* had been reported act as homo- and hetero-dimeric interactions form in Arabidopsis [48]. *KIN1* and *ERD10* were reported to encode late embryogenesis abundant (LEA)-like proteins and proposed to aid in damage repair [49,50]. In this study, the expression of *RAB18*, *ERD10*, and *KIN1* was upregulated in the *atpprt1* mutant but was downregulated in *AtPPRT1* overexpressing lines. Thus, this molecular evidence supports the phenotypical alteration between the *atpprt1* mutant and the *AtPPRT1*-overexpressing plants.

ERD10 has chaperone activity that might help protect the proteins in response to stress [51]. Accumulation of ERD10 is involved in the tolerance to subsets of abiotic stresses in Arabidopsis, and the lack of ERD10 leads to greater plant susceptibility to these stresses [52]. In this work, ERD10 in the *atpprt1* mutant was upregulated by nearly five fold compared to WT with the application of ABA, whereas there were no effects of ABA on OE lines and WT. The *atpprt1* mutant displayed enhanced tolerance to drought stress, showing no visible growth suppression by the accumulation of ERD10. It means that the AtPPRT1 loss-of-function mutant can optimize itself by the number of components involved in the stress response system.

In conclusion, the data presented here provide phenotypic and genetic evidence in support of a negative role of AtPPRT1, a novel RING domain protein, in ABA signaling. Further studies to identify E3 ligase activity and endogenous substrates of AtPPRT1 will be required to reveal the function of AtPPRT1 in abiotic stresses, as well as in normal plant growth and development.

## 4. Materials and Methods

### 4.1. Plant Material and Growth Conditions

All Arabidopsis used in this study were of Columbia (Col-0) ecotype. The T-DNA insertion mutant *atpprt1* (SALK_005268C) was obtained from the Arabidopsis Biological Resource Center (ABRC; http://abrc.osu.edu/). For nonsterile culture, all plants were grown in pots containing a mixture of vermiculite and soil (1:3, *v*/*v*) in a greenhouse at 23 ± 1 °C under 60% humidity with 16-h light (80 to 100 µE m^−2^s^−1^)/8-h dark cycles. For sterile culture, all seeds were stored for 2 days at 4 °C to break dormancy, then sterilized with NaClO (0.5% *v*/*v*) for 15 min, followed by washing with sterile water five times. Surface-sterilized seeds were sown on Murashige and Skoog (MS) medium containing 2% (*w*/*v*) sucrose and 0.8% (*w*/*v*) agar, pH 5.7. All seeds were incubated in the growth chamber at 23 ± 1 °C under a 16-h light/8-h dark photoperiod.

### 4.2. Identification of T-DNA Insertion Mutants and Generation of Transgenic Plants

The T-DNA insertion mutant *atpprt1* (SALK_005269C) was identified by genome PCR using primer pairs provided by the T-DNA primer design website (http://signalsalk.edu/tdnaprimers.2.html) (Supplementary Table S1). To generate the AtPPRT1-overexpressing and the *ProAtPPRT1::GUS* constructs, the full-length CDS of *AtPPRT1* (1407 bp) and the full-length promoter of *AtPPRT1* (1492 bp) were amplified from the cDNA of WT, and cloned into pCAMBIA1302 and pCAMBIA1301, respectively, using the One Step Cloning Kit (Vazyme, Nanjing, China). The CaMV35S promoter on pCAMBIA1301 before GUS was cut down. Each construct was introduced into *Agrobacterium tumefaciens* (GV3101) and then transformed into the wild-type Arabidopsis by the floral dip method. Transgenic plants were screened out by hygromycin resistance and mRNA levels of *AtPPRT1* were verified by qRT-PCR assays. T3 homozygous seeds of the transgenic plants were used for further analysis.

### 4.3. Phenotype Analysis

The germination rates and seedlings with green cotyledon assays were carried out as described in the reference [53]. For germination assays, the seeds grown under the same conditions were harvested from mature siliques of various plants on the same day. Then, about 150 seeds from each line were planted on solid MS medium supplemented with or without 0.3 μM ABA after 2 days stratification. Similarly, about 150 seeds from each line were planted on solid MS medium supplemented with or without 300 mM mannitol after 2 days stratification. The rates of seed germination were calculated during germination, and the rates of seedlings with green cotyledons were tracked for 7 days.

For root elongation assays, all seeds from each line were kept at 4 °C for 2 days and then sown on MS medium for vertical incubation for 3 days in the growth chamber. Then, 21 seedlings of each line sharing similar root lengths were transferred to new MS plates supplemented with or without 30 μM ABA in the vertical position. All images were collected 7 days later. All root lengths were measured and recorded using the Image J software.

For the drought-tolerance test, 27 seedlings were planted in each pot, and 4-week-old plants of each line grown under the same conditions were subjected to drought stress by withholding water for 13 days. Then, the morphological changes of all plants and the survival rates were recorded 2 days after re-watering.

For water loss rate assays, detached leaves from mature plants were placed on dishes and exposed to air in the same chamber. The weights of the detached leaves were measured every 30 min.

For stomatal aperture measurements, whole plants were placed in darkness for 24 h to close the stomata. Then, rosette leaves of 4-week-old plants were detached and submerged in stomatal opening solution (10 mM MES–KOH (pH 6.15), 10 mM KCl, and 50 μM CaCl_2_ with or without 10 μM ABA) for a 2-h exposure to continuous light. Stomatal apertures were photographed using a fluorescence microscope (DMI6000B, Leica, Germany). Length/width ratios of more than 100 stomas from each line were measured and counted using Image J.

### 4.4. Analysis of Gene Expression

Seven-day-old Arabidopsis seedlings were used for material identification and 12-day-old seedlings were used for stress-inducible and ABA hydrolysis genes expression analysis. Total RNA was extracted from different plant materials treated with or without 50 µM ABA for 4 h using RNAiso Plus reagents (Takara, Beijing, China) The cDNA was synthesized by the Prime Script RT Reagent Kit (Takara) using 1 µg RNA.

For semi-quantitative RT-PCR, semi-quantitative primers were used to test the expression of objective genes. For qRT-PCR analysis, the SYBR Premix Ex Taq Kit (Takara) and an Applied Biosystems 7500 real-time PCR system were used for the reactions according to the manufacturer’s instructions. ACTIN2 was used as the internal control for both semi-quantitative RT-PCR and qRT-PCR (primers showed in Supplementary Table S1). Parameters in qRT-PCR were performed in triplicate with the following cycling conditions: 95 °C for 5 min; 39 cycles each at 95 °C for 10 s and 50 °C for 30 s, and 72 °C for 20 s.

### 4.5. GUS Staining

The β-glucuronidase gene and pCAMBIA1301 plasmid were used as the reporter gene and effector plasmid, respectively. A proper amount of GUS dyeing solution (Real-Times Company, Detroit, Michigan, USA) was added to the test tube to completely cover the plant material. The tubes were wrapped in tinfoil and shaken for about 3 h at 37 °C until the material turned blue. Then, all material was transferred to ethanol to wash 2–3 times until the negative control material was decolored. GUS activity in different plant material was observed under a fluorescence microscope (DMI6000B, Leica, Germany).

### 4.6. Sequence Analysis of AtPPRT1

We found two homologous genes (*AT1G73950* and *AT1G18470*) of *AtPPRT1* in Arabidopsis by searching the PLAZA (https://bioinformatics.psb.ugent.be/plaza/versions/plaza_v3_dicots/). The putative protein structure of AtPPRT1 was obtained from SMART (http://smart.embl-heidelberg.de/) and NCBI (https://www.ncbi.nlm.nih.gov/protein.com). Multiple alignment results were edited in the DNAMAN 8.0 program. The phylogenetic analysis was performed with MEGA7.0 program.

### 4.7. Statistical Analysis

The data are represented as the mean ± SD. The statistical analysis was performed using the *t*-test. Values of *p* < 0.05 were considered significant, and values of *p* < 0.01 were considered more significant.

### 4.8. Subcellular Localization Assay

The *AtPPRT1* coding sequence was cloned into PBI221 containing a 35S promoter for green fluorescence protein (GFP) by homologous recombination. The pBI221-GFP was used as the control. Then, 2 µg of plastids was transformed into Arabidopsis mesophyll protoplasts. After incubation for 14 h at 22 °C, cells with GFP fluorescence was observed and captured using a confocal laser-scanning microscope (Leica TCS SP5 II system, Leica, Germany). In the same way, the PBI221-AtPPRT1-GFP was co-transformed into tobacco mesophyll protoplasts with the mitochondrial marker AtERG2 [54]. To avoid overlapping with the red fluorescence of chloroplasts, yellow fluorescence of AtERG2-RFP was observed and captured.

## 5. Conclusions

AtPPRT1, a novel protein encoded by *AT1G68820*, plays a negative role in ABA and drought stress in Arabidopsis.

## Supplementary Materials

Supplementary materials can be found at http://www.mdpi.com/1422-0067/20/2/394/s1.

## Abbreviations

OE2	*AtPPRT1*-overexpression line 2
OE10	*AtPPRT1*-overexpression line 10
PPRT1	Putative Protein with RING domain and Tmemb_185A domain
