# Supplementary material for: Expression Pattern and Function Analysis of AtPPRT1, a Novel Negative Regulator in ABA and Drought Stress Responses in Arabidopsis
Source: Int J Mol Sci. 2019 Jan 17;20(2):394. doi: 10.3390/ijms20020394 (PMC6358930; doi:10.3390/ijms20020394)
Supplement: Supplementary file 1 [file ijms-20-00394-s001.pdf]

Supplementary figure 1：

```
>userseq24037 1494nt
+ ATTAGTCGCG GTATTAAAAT TGTGCTTCCC CTTTTGGAAA AAAATAGAGT AACTGTTCAA ATCCTTATCA
- TAATCAGCGC CATAATTTTA ACACGAAGGG GAAAACCTTT TTTTATCTCA TTGACAAGTT TAGGAATAGT

+ TCCTGATTCA GGATATTAAA CCTTAAATAT TGTTTGTCAA ACTCAACTTC TATCAATCAT TTTTTATATT
- AGGACTAAGT CCTATAATTT GGAATTTATA ACAAACAGTT TGAGTTGAAG ATAGTTAGTA AAAAATATAA

+ GTTTTAATGT TAAACAACTG TCCATCTATC TTATCAACAA GAAGAAGAAT TTCTAGCCAG ACTTATAAAC
- CAAAATTACA ATTTGTTGAC AGGTAGATAG AATAGTTGTT CTTCTTCTTA AAGATCGGTC TGAATATTTG

+ TTGCACGACA AGACATTCAT CCTTTTTTAA TTAATGAACA AGAGTAACGA CTTTTACACT TCTCTTGTCA
- AACGTGCTGT TCTGTAAGTA GGAAAAAATT AATTACTTGT TCTCATTGCT GAAAATGTGA AGAGAACAGT

+ CAAAATTATT GAACAGTATT TACAAGTATT TTATAAAAAT TGCTATTGGT TGATATTTTT ATTGGTGCAT
- GTTTTAATAA CTTGTCATAA ATGTTCATAA AATATTTTTA ACGATAACCA ACTATAAAAA TAACCACGTA

+ GTCAAGATAT TTCTGGCAAC AATCATTAGA ACGCGACCAT AACATGTGCT GCAATTTGAC AATTCTTTAT
- CAGTTCTATA AAGACCGTTG TTAGTAATCT TGCGCTGGTA TTGTACACGA CGTTAAACTG TTAAGAAATA

+ ATCTAAAAAA TGACAATTTG ACCTCGTTGG AATCTTGGAT TTGTTTCTCC CAAGCACAAT ATGTTCGAGC
- TAGATTTTTT ACTGTTAAAC TGGAGCAACC TTAGAACCTA AACAAAGAGG GTTCGTGTTA TACAAGCTCG

+ TATCGTACCA TTTCTGAAAG TGTAATTATA AAGACTGCAA TCATATATGG GCTCGTGGAG CTAAATCATG
- ATAGCATGGT AAAGACTTTC ACATTAATAT TTCTGACGTT AGTATATACC CGAGCACCTC GATTTAGTAC

+ GCCTAAAGTT TTTTTTTAAT CAAATGGATT TTGAACTTGT GGTAAAGTTT TCGGATCTAC GAAAAATTAT
- CGGATTTCAA AAAAAAATTA GTTTACCTAA AACTTGAACA CCATTTCAAA AGCCTAGATG CTTTTTAATA

+ GCATTCTTAA TCAAACCTCT AATTCGATAA GACAATTGTT TTCCAACAAA GAATGTGTCA AAACTCAAAA
- CGTAAGAATT AGTTTGGAGA TTAAGCTATT CTGTTAACAA AAGGTTGTTT CTTACACAGT TTTGAGTTTT

+ ACTCTTAACA ATTGTTTTGA ATGTTTTCCA ATAATACCAC ATCGGTTTAT GTATCAATTA ATATTCCAAA
- TGAGAATTGT TAACAAAACT TACAAAAGGT TATTATGGTG TAGCCAAATA CATAGTTAAT TATAAGGTTT

+ AGTTGATACT CCCAACGATA TTCCTATGAC CTAGAATTTG ATCCATATCT CAAAATTTTA CGACCGACAA
- TCAACTATGA GGGTTGCTAT AAGGATACTG GATCTTAAAC TAGGTATAGA GTTTTAAAAT GCTGGCTGTT

+ ATATATTAAT GTTCTTTTAT TTTATTTTAT ATAGATTAAT GGTTTAGTCA TCTACATGTA TTCATTAATA
- TATATAATTA CAAGAAAATA AAATAAAATA TATCTAATTA CCAAATCAGT AGATGTACAT AAGTAATTAT

+ CACCTATATA CTGTAATATT TTCACGAAAC TTTTAAACAT TTACAAAAAA TGAAGAGATT AACTTTGAAA
- GTGGATATAT GACATTATAA AAGTGCTTTG AAAATTTGTA AATGTTTTTT ACTTCTCTAA TTGAAACTTT

+ CGGAAGAGTG TTAATTACTT AATTATGTAT GTTTTGTTTG TTTGTCAACG AAGAGTATTA TTTTTTTATA
- GCCTTCTCAC AATTAATGAA TTAATACATA CAAAACAAAC AAACAGTTGC TTCTCATAAT AAAAAAATAT

+ TACTAATATG AAGACAAAAC ATATAATTCC ATGTCACCTC GTAATCTCGT ATGTTGTAAA AAAAATATCT
- ATGATTATAC TTCTGTTTTG TATATTAAGG TACAGTGGAG CATTAGAGCA TACAACATTT TTTTTATAGA
+ CTATCCAATA GTATTTCACT ACCTAGTTTA AATCGCATTC TCTATAATAC GTTACCATAT TCCCCAAATT
- GATAGGTTAT CATAAAGTGA TGGATCAAAT TTAGCGTAAG AGATATTATG CAATGGTATA AGGGGTTTAA

+ ACCCTTAGCT ATGACCATAG TCGCTGGCTC GCTGCAAAAA GTGAAAGGGG TATATAGGTA AAAAAGGATA
- TGGGAATCGA TACTGGTATC AGCGACCGAG CGACGTTTTT CACTTTCCCC ATATATCCAT TTTTTCCTAT

+ AATTAACAGA GAGAGGAAGG GCTTTGGCGT GGAAGCTCAG CTTTTCTCCG AGTCGGGAAA ATGTCTTTTT
- TTAATTGTCT CTCTCCTTCC CGAAACCGCA CCTTCGAGTC GAAAAGAGGC TCAGCCCTTT TACAGAAAAA

+ CTCGAAAGAA TAAATCTTTT TCTCTTCTCT CTGTGTTACC CTCTTGAAAT TTTCCGCGAA AAATCTCAAC
- GAGCTTTCTT ATTTAGAAAA AGAGAAGAGA GACACAATGG GAGAACTTTA AAAGGCGCTT TTTAGAGTTG

+ TTTTCCATCT GAAAACCCCC CAAAATCAAA CAATTGGATC TTGATTTTTA GCTGTGATTT AGTGATTAAG
- AAAAGGTAGA CTTTTGGGGG GTTTTAGTTT GTTAACCTAG AACTAAAAAT CGACACTAAA TCACTAATTC

+ AGGCTTAGGG TTTGTGGCGA AAT
- TCCGAATCCC AAACACCGCT TTA
```

MBS

G-BOX

HSE

Supplementary figure 1： The sequence analysis of AtPPRT1 promoter. The promoter sequence was obtained from TAIR (https://www.arabidopsis.org/). The cis-acting elements on the promoter were analysed on PlantCARE (http://bioinformatics.psb.ugent. be/webtools/plantcare/html/).

Supplementary figure 2：

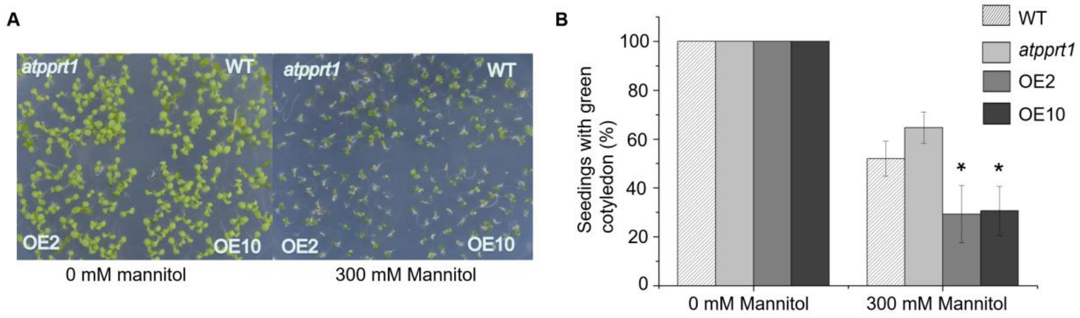

Supplementary figure 2. AtPPRT1 plays a negative role in the response of Arabidopsis to osmotic stress.(A) and (B) Phenotype and green cotyledon rates of WT, atpprt1, OE2 and OE10 grown 7 days on MS or MS supplemented with 300 mM Mannitol. The values are the average of three individual biological replications. Error bars represent ± SDs (n=50, *P < 0.05 and **P < 0.01, t-test).

Supplementary table 1:

35S-AtPPRT1-F	ACGGGGGACTCTTGACATGTTGGTACAGAGAAGAG
35S-AtPPRT1-R	ACTAGTCAGATCTACCATCACATCGTATACAGGCATACGC
pAtPPRT1-F	TTCGAGCTCGGTACCCGATTAGTCGCGGTATTAAAATTGTG
pAtPPRT1-R	TACCCTCAGATCTACCATTTCGCCACAAACCCTAAGCCTCTTA
35S-F	GACGCACAATCCCACTATCC
GUS-R	CATTGTTTGCCTCCCTGCTGC
LP	TGCAGGTTGTCTCTGACATTG
RR	AAAGAATCCCAGGAATTCACG
LBb1.3	ATTTTGCCGATTTCGGAAC
actin-F	ACATCCCACCTACTGGTCTGAAG
actin-R	GCATCTTGGTATTGCTGGTACTCT
KIN1-F	TCAGAGACCAACAAGAATG
KIN1-R	TTGTCCAGCAGAACATTG
RAB18-F	CAGCAGCAGTATGACGAGTA
RAB18-R	CAGTTCCAAAGCCTTCAGTC
ERD10-F	TCTCTGAACCAGAGTCGTTT
ERD10-R	CTTCTTCTCACCGTCTTCAC
CYP707A1-F	TGGCTCCAAAACCCAATACGT
CYP707A1-R	CGAATGGCCCATACTGAATC
CYP707A3-F	TGGTAGTGGGATTCATTCTTGT
CYP707A3-R	TACGATTGACCATCTGTACTTAGT
AtPPRT1-221-F	AGAGAACACGGGGGACTCTAGAATGTTGGTACAGAGAAG
AtPPRT1-221-R	GACCACCCGGGGATCCTCTAGACACATCGTATACAGGCA
ATERG2-RFP-F	CGCGGATCCATGAAAGCTTTTAGAT
ATERG2-RFP-R	ACGCGTCGACCCAGGTTAAGCTCAAG

Supplementary table 1: Primers used in experiments.
